# Supplementary material for: All-at-once RNA folding with 3D motif prediction framed by evolutionary information
Source: Nat Methods. 2025 Oct 3;22(10):2094–106. doi: 10.1038/s41592-025-02833-w (PMC12510878; doi:10.1038/s41592-025-02833-w)
Supplement: Supplementary file 3 — Source code to run CaCoFold-R3D. Data and results. [file 41592_2025_2833_MOESM3_ESM.gz › supplemental_material/results/CaCoFold-R3D/CaCoFold-R3D_Rfam/RF02155_NCRUPAR_2.R2R.sto.pdf]

RF02155_NCRUPAR_2

5′- A Y R Y ● A A A G A G U A A A C U C A A A G A A A G U A G A G G A A A A G A A U G A A U U A A A R A U Y U G U ○ ○ U ● A U A A A U A A A A A U A A A A G U A A Y R G A R C A A A A A G A Y G G G C A U
